# Clinical outcomes of patients with T4 or N1b well-differentiated thyroid cancer after different strategies of adjuvant radioiodine therapy

**DOI:** 10.1038/s41598-019-42083-3

**Published:** 2019-04-03

**Authors:** Shin Young Jeong, Sang-Woo Lee, Wan Wook Kim, Jin Hyang Jung, Won Kee Lee, Byeong-Cheol Ahn, Jaetae Lee

**Affiliations:** 1 Department of Nuclear Medicine, School of Medicine, Kyungpook National University, Daegu, Republic of Korea; 2 Department of Breast and Thyroid Surgery, School of Medicine, Kyungpook National University, Daegu, Republic of Korea; 3 Medical Research Collaboration Center in KNUH, School of Medicine, Kyungpook National University, Daegu, Republic of Korea; 4 Department of Nuclear Medicine, Kyungpook National University Chilgok Hospital, Daegu, Republic of Korea; 5 Department of Breast and Thyroid Surgery, Kyungpook National University Chilgok Hospital, Daegu, Republic of Korea; 6 Department of Nuclear Medicine, Kyungpook National University Hospital, Daegu, Republic of Korea

## Abstract

We aimed to determine whether recombinant human thyrotropin (rhTSH) plus 3.7 GBq could replace thyroid hormone withdrawal (THW) plus 5.55 GBq for adjuvant radioactive iodine (RAI) therapy in differentiated thyroid cancer (DTC) patients with T4 or N1b disease. This study was a retrospective study comparing ablation success rate, response to initial therapy, and recurrence-free survival (RFS) of patients with rhTSH plus 3.7 GBq versus those with THW plus 5.55 GBq in 253 DTC patients with T4 or N1b disease. There were no differences in the TSH-stimulated thyroglobulin level, rate of incomplete response after initial treatment, or the RFS between the two treatment strategies. However, thyroid bed uptake on follow-up diagnostic RAI whole-body scanning (WBS) was more frequently observed in the group treated with rhTSH plus 3.7 GBq than in the group with THW plus 5.55 GBq. Adjuvant RAI therapy with rhTSH plus 3.7 GBq had comparable results in the absence of persistent tumor, compared with that with THW plus 5.55 GBq. Although thyroid bed uptake was more frequently observed, rhTSH plus 3.7 GBq may be used instead of THW plus 5.55 GBq for adjuvant RAI therapy in patients with T4 or N1b disease.

## Introduction

Postoperative radioactive iodine (RAI) therapy has been used for patients with differentiated thyroid cancer (DTC) to remove residual normal thyroid tissue after thyroidectomy (remnant ablation), or to treat potential metastatic disease (adjuvant therapy). Low-risk patients are administered a low activity of ^131^I to achieve remnant ablation for the purpose of facilitating follow-up. In patients with locally advanced disease (T4) or extensive lymph node disease (N1b) who are at significant risk of potential micrometastasis, a high dose of ^131^I is routinely administered for tumoricidal effect as an adjuvant therapy, not merely for remnant ablation. However, the optimal dose and preparation of RAI therapy remain controversial^1^. In adjuvant RAI therapy, it is uncertain whether routine use of higher administered activities will reduce structural disease recurrence for T4 and N1b disease. Furthermore, there are few data from controlled long-term outcome studies to show that recombinant human thyrotropin alfa (rhTSH) preparation may be considered as an alternative to thyroid hormone withdrawal (THW) prior to adjuvant RAI therapy for T4 and N1b disease.

Our institution has used THW plus 5.55 GBq (150 mCi) for adjuvant RAI therapy in patients with T4 or N1b disease. There is little evidence to suggest that increasing administered activities of adjuvant RAI is necessarily associated with improvement in clinical outcomes for patients with American Thyroid Association (ATA) intermediate- and high-risk disease without evidence of persistent disease. RAI ablation/adjuvant therapy may induce damage to lacrimal and salivary glands, depending on the amount of radioactivity administered^2–4^. The use of rhTSH maintains quality of life^5^ and reduces the radiation activity delivered to the body as compared with THW^6^. In ATA low risk disease, the rate of ablation success with an administered activity of 1.11 GBq (30 mCi) was reported to be non-inferior compared to 3.7 GBq (100 mCi) after preparation with THW or rhTSH^7,8^. Hugo *et al*. reported rhTSH preparation for RAI remnant ablation can be effectively used in intermediate and high-risk patients without known distant metastasis^9^. Furthermore, national health insurance of the Republic of Korea has covered the use of rhTSH for remnant ablation and adjuvant therapy if the activity of RAI is less than 3.7 GBq. Therefore, as of October 2013, we changed the strategy of adjuvant RAI therapy in our institution to rhTSH plus 3.7 GBq instead of THW plus 5.55 GBq in patients with T4 or N1b thyroid cancer, to achieve better quality of life for patients and lower rates of adverse events.

In this study, we aimed to determine whether rhTSH plus 3.7 GBq of RAI could be used instead of THW plus 5.55 GBq of RAI for adjuvant RAI therapy in patients with T4 or N1b disease.

## Methods

### Patients

This study was a retrospective study comparing the thyroid remnant ablation success of rhTSH plus 3.7 GBq of RAI versus THW plus 5.55 GBq of RAI in DTC patients with T4 or N1b disease. The data collection and analysis were approved by Institutional Review Board of Kyungpook National University Chilgok Hospital, and written informed consent was waived.

We performed a retrospective review of 1307 DTC patients who had undergone RAI ablation or adjuvant therapy between January 2011 and June 2015. Eligibility criteria were an age of 18 to 80 years and histological confirmation of DTC with T4 or N1b disease requiring adjuvant RAI therapy, but with no distant metastasis and no microscopic residual disease observed after total thyroidectomy with central and/or lateral lymph-node dissection.

Exclusion criteria were the presence of aggressive malignant variants, including tall-cell, insular, poorly differentiated, and diffuse sclerosing thyroid cancer; anaplastic or medullary carcinoma; pregnancy; severe coexisting conditions (including severe heart failure and renal failure); previous cancer with limited life expectancy; antithyroglobulin antibody positivity; and previous treatment for thyroid cancer, with the exception of surgery. Finally, 253 PTC patients were selected for the current study. Among the 253 patients, 125 patients were treated with THW plus 5.55 GBq from January 2011 to September 2013, and 128 patients received rhTSH plus 3.7 GBq from October 2013 to June 2015.

### Adjuvant RAI therapy and assessments

Before the commencement of adjuvant RAI therapy, patients followed a low-iodine diet for 2 weeks and achieved endogenous or exogenous thyrotropin elevation. In the group undergoing THW, at least 3 weeks of levothyroxine withdrawal was performed, and on the day of ^131^I administration, thyroglobulin and thyrotropin were measured to check that their TSH levels exceeded an empirical 30 mIU/L, a prespecified cutoff value that was required for adjuvant RAI therapy. In the thyrotropin alfa group, rhTSH (Thyrogen, Genzyme) was administered during treatment with thyroid hormone, at an activity of 0.9 mg intramuscularly on 2 consecutive days, and RAI was administered on the next day after the second injection. Blood was obtained to measure thyroglobulin and thyrotropin approximately 24 hours after the second injection. Whole-body scan (WBS) was performed 5 to 7 days after administration of ^131^I using a dual-head c-camera equipped with 1.59 cm (5/8 in) NaI crystals and a multidetector (16-row) spiral CT (NM670; General Electric Medical Systems, Milwaukee, WI, USA).

After total thyroidectomy, followed by adjuvant RAI therapy, all patients were started on thyroid hormone-suppression therapy and Tg levels were measured every 6–12 months. Neck ultrasonography (USG), TSH-stimulated thyroglobulin or TSH-suppressed thyroglobulin, anti-thyroglobulin antibody, and diagnostic WBS were used for surveillance of response to initial therapy and recurrence. Neck USG was performed every 6 months or 1 year, and fine-needle aspiration cytology was performed if there were any suspicious features on neck USG. The level of TSH-stimulated thyroglobulin was determined after levothyroxine withdrawal according to an elevated TSH level more than 30 mIU/L. The acquisition of diagnostic WBS was performed 1 day after ^123^I (185 MBq, 171 patients) administration or 2–3 days after ^131^I (111 MBq, 82 patients) administration. Thyroglobulin and thyroglobulin antibody were measured by radioimmunoassay (Thyroglobulin IRMA; CIS Bio International, Gif Sur Yvette, France; Brahms anti-Tgn radioimmunoassay; BRAHMS GmbH, Hennigsdorf, Germany).

### Study endpoints

The primary endpoint was the rate of ablation success, which was determined in 3 ways, using TSH-stimulated thyroglobulin level, neck USG findings, and diagnostic WBS results. The cut-off value of TSH-stimulated thyroglobulin for ablation success was less than 1 ng/mL at 6 to 18 months after adjuvant RAI therapy. The first set of criteria for ablation success included only the TSH-stimulated thyroglobulin level, while the second considered the TSH-stimulated thyroglobulin level and neck USG findings, regardless of the diagnostic WBS results. The final set of criteria for ablation success included diagnostic WBS results along with TSH-stimulated thyroglobulin level and neck USG findings.

Secondary endpoints were response to initial therapy and recurrence. The response to initial therapy was classified according to the 2015 ATA management guidelines for adult patients with thyroid nodules and DTC 6–12 months after RAI adjuvant therapy: (i) excellent response if patients achieved a TSH-stimulated thyroglobulin level <1 ng/dL and had a normal neck USG and/or no visible uptake on diagnostic WBS; (ii) indeterminate response if patients achieved a TSH-stimulated thyroglobulin level between 1 and 10 ng/dL and had nonspecific findings on neck USG and/or bed uptake on diagnostic WBS; (iii) biochemical incomplete response if patients had a TSH-stimulated thyroglobulin level >10 ng/dL and no findings or nonspecific findings on neck USG or cross-sectional imaging; or (iv) structural incomplete response if patients had suspicious findings on neck USG or cross-sectional imaging or had abnormal uptake on follow-up diagnostic WBS (not thyroid bed uptake) with any TSH-stimulated thyroglobulin level^1^. Diagnostic WBS was classified as no uptake, bed uptake or pathologic uptake according to location of hot uptake. Neck USG findings were classified as negative, nonspecific or suspicious findings. The classification of neck USG and diagnostic WBS was according to medical records.

Recurrence-free survival (RFS) was defined as the time from treatment completion to tumor recurrence. To evaluate recurrence, physical examination was performed every 3 months for 2 years, and then every 6 months from 2–5 years, and annually thereafter. Imaging studies and biochemical evaluation were performed every 6–12 months and/or when the studies were clinically indicated. Recurrence was defined as a newly detected, cytologically or pathologically confirmed lesions, and the clinical endpoint was considered the time of recurrence or the end of follow-up.

### Statistical analysis

Descriptive quantitative data are expressed as means ± standard deviation (SD); qualitative data are expressed as percentages. Significant differences between groups were ascertained by means of independent T-test/ Mann–Whitney U test (for quantitative variables) or a chi-square test (for qualitative variables). The differences in observed ablation success rates and their 95% bilateral confidence intervals (95% CI) are presented. Survival curves for RFS were constructed using the Kaplan-Meier method.

Statistical analyses were performed with the use of SPSS software version 22.0 (SPSS, Chicago, IL, USA), the MedCalc® statistical package (version 17.9.6, MedCalc Software bvba, Ostend, Belgium) and SAS version 9.4 (SAS Institute Inc, Cary, NC, USA). All *P*-values were two-sided and *P*-values less than 0.05 were considered statistically significant.

### Ethical standards

All procedures performed in studies involving human participants were in accordance with the ethical standards of the institutional and/or national research committee and with the 1964 Helsinki declaration and its later amendments or comparable ethical standards. Due to our retrospective review of the data, and the requirement of an informed consent was waived after approval of our institutional review board.

## Results

### Patient characteristics

The clinicopathological characteristics of the 253 patients included in this study are summarized in Table 1. We included only patients with DTC with pathologic T4 or N1b disease without clinical evidence of distant metastasis at the time of thyroid cancer diagnosis. Most were women (77%) presenting with small primary tumors (median 1.4 cm; range 0.1–8.5 cm) with extrathyroidal extension (86%). Most of the primary tumors were papillary thyroid cancer. Consistent with the American Joint Committee on Cancer (AJCC)/TNM staging system (7^th^ edition), all patients were classified as either Stage I (<45 years old at diagnosis, 45%) or Stage IVa (>45 years old at diagnosis, 55%). According to the 2015 ATA management guidelines, 110 patients (43.5%) were classified as high-risk disease and 143 patients (56.5%) were classified as intermediate-risk disease. Those who had high risk features on pathologic examination were as follows: 82 patients had macroscopic gross extrathyroidal extension, 24 patients had pathologic N1 with any metastatic lymph node >3 cm in largest dimension, 15 patients had postoperative serum thyroglobulin suggestive of distant metastases, or 1 patient had follicular thyroid cancer with extensive vascular invasion (>4 foci of vascular invasion). In patients with N1b disease, 33 patients had macroscopic gross extrathyroidal extension, 24 patients had pathologic N1 with any metastatic lymph node >3 cm in largest dimension, 14 patients had postoperative serum thyroglobulin suggestive of distant metastases (greater than 10 ng/mL)^1^, or 1 patient had follicular thyroid cancer with extensive vascular invasion (>4 foci of vascular invasion).

**Table 1:** Baseline clinicopathological characteristics of study subjects.

	Total	THW plus 5.55 GBq	rhTSH plus 3.7 GBq	T4		N1b	
				THW plus 5.55 GBq	rhTSH plus 3.7 GBq	THW plus 5.55 GBq	rhTSH plus 3.7 GBq
Patients M/F	253(59/194)	125(28/97)	128(31/97)	38(6/32)	44(11/33)	97(23/74)	104(28/76)
		*p* = 0.768		*p* = 0.415		*p* = 0.630	
Age (years)	46.4 ± 13.4	47.0 ± 13.6	45.8 ± 13.2	51.7 ± 15.1	48.6 ± 14.7	45.2 ± 13.0	45.0 ± 13.1
		*p* = 0.497		*p* = 0.363		*p* = 0.904	
Histology PTC/FTC	252/1	124/1	128/0	37/1	44/0	96/1	104/0
		*p* = 0.494		*p* = 0.463		*p* = 0.483	
Tumor size (cm)	1.65 ± 1.34	1.50 ± 0.93	1.80 ± 1.63	1.74 ± 1.08	1.73 ± 1.02	1.50 ± 0.97	1.89 ± 1.75
		*p* = 0.068		*p* = 0.982		*p* = 0.058	
TNM stage Stage I/IV	114/139	58/67	40/72	12/26	18/26	49/48	47/57
		*p* = 0.369		*p* = 0.491		*p* = 0.470	
AJCC T stage T1/T2/T3/T4	27/6/138/82	21/4/62/38	6/2/76/44			20/4/60/13	6/2/76/20
		*p* = 0.056				*p* = 0.063	
AJCC N stage N0/N1a/N1b/Nx	15/34/201/3	6/20/97/2	9/14/104/1	6/17/13/2	9/14/20/1		
		*p* = 0.532		*p* = 0.516			
sTg at ablation	2.54 ± 6.10	3.06 ± 6.67	2.03 ± 5.46	2.33 ± 3.35	2.86 ± 8.70	3.31 ± 7.36	2.27 ± 6.01
		*p* = 0.181		*p* = 0.721		*p* = 0.274	

A total of 125 patients were treated with THW plus 5.55 GBq, and 128 patients received rhTSH plus 3.7 GBq. There were no significant differences in sex, age, histologic type, tumor size, TNM stage, or TSH-stimulated thyroglobulin value at adjuvant RAI therapy between the two groups (Table 1).

### Ablation success

The ablation success rates were 63.2–72.0% in the patients treated with THW plus 5.55 GBq and 46.9–70.3% in the patients treated with rhTSH plus 3.7 GBq, depending on the criteria used to determine successful ablation (Table 2). Ablation was successful in 90 of the 125 patients (72.0%) treated with THW plus 5.55 GBq versus 90 of the 128 patients (70.3%) treated with rhTSH plus 3.7 GBq, as determined only by TSH-stimulated thyroglobulin level 6–18 months after adjuvant RAI therapy (Table 2). The difference in the ablation success rate for this comparison was −1.7 percentage points (95% CI: −0.13 to 0.09).

**Table 2:** Ablation success after THW plus 5.55 GBq and rhTSH plus 3.7 GBq radioactive iodine therapy according to various criteria.

Criteria for ablation success	THW plus 5.55 GBq n (%)	rhTSH plus 3.7 GBq n (%)	Mean Difference for ablation success rate % (95% CI)	*P*-value
s-Tg <1 ng/mL, negative TgAb	90 (72.0%)	90 (70.3%)	−1.7% (−0.13 to 0.09)	0.903
s-Tg <1 ng/mL, negative TgAb no suspicious finding on USG	83 (66.4%)	79 (61.7%)	−4.7% (−0.16 to 0.07)	0.611
s-Tg <1 ng/mL, negative TgAb no suspicious findings on USG no residual thyroid bed uptake on diagnostic RAI WBS	79 (63.2%)	60 (46.9%)	−16.3% (−0.28 to −0.04)	0.019

Ablation success was determined using TSH-stimulated thyroglobulin level, neck USG findings, and diagnostic RAI whole body scan results at 6 to 18 months after adjuvant RAI therapy. Levothyroxine withdrawal was used for TSH-stimulation.

As determined by both TSH-stimulated thyroglobulin level and neck USG results, ablation was successful in 83 of the 125 patients (66.4%) treated with THW plus 5.55 GBq versus 79 of the 128 patients (61.7%) treated with rhTSH plus 3.7 GBq (Table 2). The difference in the ablation success rate for these comparisons was −4.7 percentage points (95% CI: −0.16 to 0.07).

However, ablation was successful in 79 of the 125 patients (63.2%) treated with THW plus 5.55 GBq versus only 60 of the 128 patients (46.9%) treated with rhTSH plus 3.7 GBq, as determined by TSH-stimulated thyroglobulin level, neck USG, and diagnostic RAI WBS results (Table 2). The difference in the ablation success rate for these comparisons was −16.3 percentage points (95% CI: −0.28 to −0.04). This result is caused by a higher number of the patients with residual thyroid bed uptake on a diagnostic RAI WBS (34/128, 26.5% vs 7/125, 5.6%), not extrathyroidal pathologic uptake, in the group treated with rhTSH plus 3.7 GBq than those using THW plus 5.55 GBq. There was no difference in rate of patients with bed uptake on diagnostic whole body scan between ^123^I (28/171 patients, 16.4%) and ^131^I (13/82 patients, 15.9%). The subgroup analysis with older patients (>45years) also showed significant difference in percentage of residual thyroid bed uptake on diagnostic WBS between adjuvant RAI therapy strategies. Five of 67 patients (7.5%) treated with THW plus 5.55 GBq showed residual thyroid bed uptake on diagnostic WBS, whereas 18 of 63 patients (28.6%) treated with rhTSH plus 3.7 GBq showed residual thyroid bed uptake on diagnostic WBS (Supplemental Table 1).

There were no significant differences of the ablation success rates between the THW plus 5.55 GBq and the rhTSH plus 3.7 GBq on the basis of TSH-stimulated thyroglobulin level and/or neck USG results (*P* = 0.611, *P* = 0.903, respectively), but ablation success rates differed significantly between the THW plus 5.55 GBq and the rhTSH plus 3.7 GBq as determined by TSH-stimulated thyroglobulin level, neck USG, and diagnostic WBS results (*P* = 0.019).

### Response to initial therapy and recurrence-free survival

Neck USG findings and TSH-stimulated thyroglobulin test performed at 6–18 months after adjuvant RAI therapy did not differ between patients who received with the THW plus 5.55 GBq and those who received rhTSH plus 3.7 GBq (Table 3). In analysis of response to initial therapy, 23 of 125 patients (18.4%) treated with THW plus 5.55 GBq showed biochemical or structural incomplete response, and 23 of 128 (18.0%) patients treated with rhTSH plus 3.7 GBq showed biochemical or structural incomplete response. Structural incomplete response was observed in 13 patients (10.4%) treated with THW plus 5.55 GBq, and in 18 patients (14.1%) treated with rhTSH plus 3.7 GBq. There was no significant difference in this response between the two groups according to adjuvant RAI therapy strategies. In subgroup analysis with old patients (>45 years), there was no significant difference in structural incomplete response between the two groups according to adjuvant RAI therapy strategies (9.0% patients treated with THW plus 5.55 GBq versus 9.5% patients treated with rhTSH plus 3.7 GBq) (Supplemental Table 1). However, RAI uptake in thyroid bed on diagnostic WBS was observed in 34 of 128 patients (26.5%) treated with rhTSH plus 3.7 GBq, but in only 7 of 125 patients (5.6%) treated with THW plus 5.55 GBq (Table 3). Furthermore, the percentage of excellent response was decreased in patients treated with rhTSH plus 3.7 GBq (43.0%), compared to that observed in those treated with THW plus 5.55 GBq (60.8%). This difference was attributed to the difference in diagnostic WBS findings.

**Table 3:** Clinical findings and classification of response to therapy after 5.55 GBq by THW and 3.7 GBq by rhTSH radioactive iodine therapy.

	THW plus 5.55 GBq	rhTSH plus 3.7 GBq
**sTg at F/U (ng/mL)**	2.00 ± 3.84	1.86 ± 5.56
	*p* = 0.816	
Neck USG		
negative	105 (84.0%)	108 (84.4%)
nonspecific	9 (7.2%)	4 (3.1%)
suspicious	11 (8.8%)	16 (12.5%)
	*p* = 0.240	
Diagnostic RAI WBS		
no uptake	116 (92.8%)	94 (73.4%)
bed uptake	7 (5.6%)	34 (26.5%)
pathologic uptake	2 (1.6%)	0 (0%)
	*p* < 0.001	
Classification of Response to Treatment		
Excellent	76 (60.8%)	55 (43.0%)
Indeterminate	26 (20.8%)	50 (39.1%)
Biochemical Incomplete	10 (8.0%)	5 (3.9%)
Structural Incomplete	13 (10.4%)	18 (14.1%)
	*p* = 0.004	
**Recurrence**	13 (10.4%)	9 (7.0%)
	*p* = 0.936*	

TSH-stimulated thyroglobulin measurement, neck USG, and diagnostic RAI whole body scan were performed at 6 to 18 months after adjuvant RAI therapy under levothyroxine withdrawal.

THW, thyroid hormone withdrawal; rhTSH, recombinant human thyrotropin; sTg at F/U, TSH-stimulated thyroglobulin level that measured at 6–18 months after initial adjuvant RAI therapy; USG, neck ultrasonography; Diagnostic RAI WBS, diagnostic radioactive iodine whole-body scan; *P value was obtained by Logrank test.

**Figure 1:**
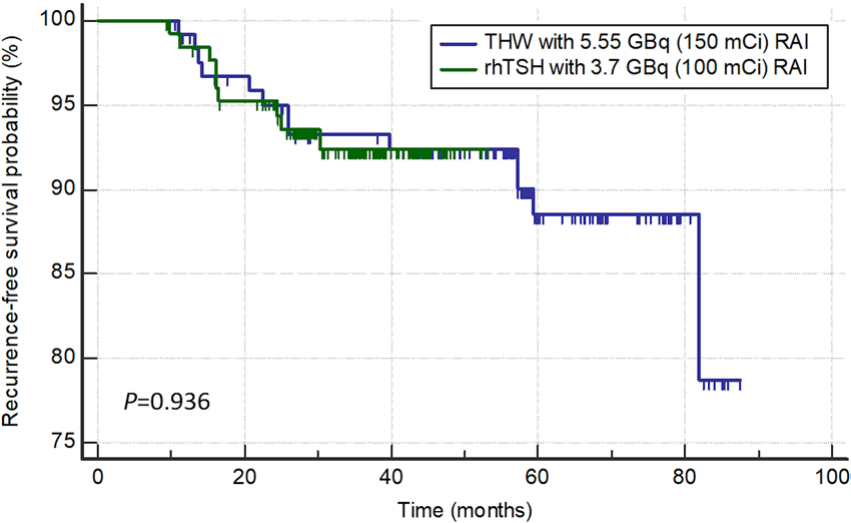
Kaplan-Meier analysis of recurrence-free survival according to adjuvant radioactive iodine (RAI) therapy strategies; THW plus 5.5 GBq of RAI and rhTSH plus 3.7 GBq of RAI.

During the mean follow up period of 43.6 ± 19.4 months (THW plus 150 mCi: 57.3 ± 20.1 months; rhTSH plus 100 mCi: 34.4 ± 9.6 months), 22 patients (8.7%) experienced disease recurrence. Recurrence was observed in 13 patients treated with THW plus 5.55 GBq (10.4%) and 9 patients treated with rhTSH plus 3.7 GBq (7.0%) and all cases were diagnosed postoperatively as a result of cervical lymph node metastasis except one case with lung metastasis. Logrank test indicated that RFS did not differ significantly according to adjuvant RAI therapy strategies (*P* = 0.936, Fig. 1). Logrank test in older patients (>45 years) also demonstrated that RFS did not differ significantly according to adjuvant RAI therapy strategies (*P* = 0.397).

## Discussion

Because of the high risk of structural disease recurrence and concern for the possibility of distant metastases, most clinicians continue to recommend RAI ablation/adjuvant therapy for patients with DTC presenting with locally advanced disease or extensive lymph node metastases, even though the data concerning clinical benefits are conflicting^1,10^. Despite the fact that adjuvant RAI therapy is usually recommended for T4 and N1b disease, there is little consensus regarding the TSH elevation method and the optimal administered activity of ^131^I.

In this study, there was no significant difference of the ablation success rate between two groups using TSH-stimulated thyroglobulin and/or neck USG. However, when the diagnostic WBS findings were taken into consideration in terms of the ablation success, the ablation success rate of rhTSH plus 3.7 GBq was significantly decreased, compared to those of THW plus 5.55 GBq. In a similar trend, there were no significant differences in TSH-stimulated thyroglobulin level and neck USG findings between the two groups in the initial treatment evaluation, but remnant bed uptake on diagnostic WBS was more frequently observed in the group receiving rhTSH plus 3.7 GBq than in the group receiving THW plus 5.55 GBq. Considering these results, it can be seen that incomplete ablation of the thyroidectomy bed occurs more frequently with the use of rhTSH plus 3.7 GBq for adjuvant RAI therapy in T4 and N1b disease.

Two factors, the dosage of RAI and the preparation method, were considered as potential factors involved in the frequent observation of remnant bed uptake on diagnostic WBS. First, insufficient RAI dosage for the volume of remnant thyroid tissue might be one of the reasons for the frequent observation of residual thyroid bed uptake. Higher radioactivity of RAI is more effective for the ablation of large normal-thyroid remnants and the eradication of micrometastases^11,12^. However, low radioactivity may be non-inferior for ablation of small normal thyroid remnants after total thyroidectomy^12^. The present study included only patients operated on by two experienced, high-volume surgeons (W.W.K. and J.H.J.). There were no differences in surgeons between the two groups, and the two surgeons, with more than 10 years of thyroid surgery experience, did not leave large amounts of remnant tissue. Furthermore, there is little evidence to suggest that increasing administered activities of adjuvant RAI is necessarily associated with improvement in clinical outcomes for patients with ATA intermediate- and high-risk disease without evidence of persistent disease.

Second, the use of rhTSH instead of THW may contribute to the residual thyroid bed uptake on diagnostic WBS. van der Horst-Schrivers *et al*. reported the relationship between use of rhTSH and visible uptake on diagnostic WBS^13^. They found that 6 out of 17 patients showed visible uptake on diagnostic WBS in the original thyroid bed on ^131^I post-therapy WBS, when rhTSH plus 3.7 GBq of RAI was used for remnant ablation after total thyroidectomy in patients with DTC. Successful ablation was also observed in only 9 out of 17 patients (53%) using the stricter definition of successful ablation (no visible uptake in the thyroid bed and a stimulated thyroglobulin level of <1 ng/mL). In contrast, Bartenstein *et al*. reported that the use of rhTSH as preparation for thyroid remnant ablation in patients with T4 primary tumors achieved a high ablation success rate that was non-inferior to that observed after THW^14^.

Previous studies showed higher ablation dose may offer some benefit to older patients^15,16^. However, subgroup analysis with older patients showed similar results with all patients. Logrank test in older patients indicated that RFS did not differ significantly according to adjuvant RAI therapy strategies, and there was no significant difference in structural incomplete response between the two groups. Similarly to the results of entire patient population, there was significant difference in percentage of residual thyroid bed uptake on diagnostic WBS between adjuvant RAI therapy strategies. Five of 67 patients (7.5%) treated with THW plus 5.55 GBq showed residual thyroid bed uptake on diagnostic WBS, not extrathyroidal pathologic uptake, whereas 18 of 63 patients (28.6%) treated with rhTSH plus 3.7 GBq showed residual thyroid bed uptake on diagnostic WBS (*P* = 0.004).

Few studies on the clinical significance of remnant bed uptake on diagnostic WBS after ablation/adjuvant RAI therapy have been reported. Lim *et al*. reported on the clinical significance according to administration of additional RAI treatment in patient with DTC with thyroid bed uptake on diagnostic WBS despite undetectable thyroglobulin levels^17^. They found no remarkable differences in clinical outcomes between observation and treatment groups of patients with DTC with thyroid bed on diagnostic WBS despite undetectable thyroglobulin levels. During a follow-up period of 53.2 ± 10.1 months, recurrence was observed in only 2 of 44 patients, suggesting the need for additional large scale controlled studies.

The advantages of the use of rhTSH over THW are well described in two large randomized trials^7,8^. They found that the proportion of patients with symptoms of hypothyroidism such as difficulty concentrating, fatigue, puffy face and hands, or sleep disturbance was significantly higher in the groups undergoing THW than in the groups receiving rhTSH. Borget *et al*. also reported that THW caused a clinically significant deterioration of health-related quality-of-life, whereas it remained stable with rhTSH^18^. Previously, we evaluated the changes in salivary gland function after RAI ablation in patients with DTC by salivary gland scintigraphy and related xerostomia symptoms^4^. Scintigraphic changes and subjective symptoms of salivary dysfunction were observed in dose-dependent manner between the patients receiving 5.55 GBq and 3.7 GBq. Considering the lower rates of adverse events, rhTSH plus 3.7 GBq may be used instead of THW plus 5.55 GBq for adjuvant RAI therapy in patients with T4 or N1b disease, although thyroid bed uptake on diagnostic WBS was more frequently observed in patients receiving rhTSH plus 3.7 GBq than in those with THW plus 5.55 GBq.

There are some limitations to the current study. The first limitation is the retrospective study design. However, bias was minimized by applying different adjuvant RAI therapy strategies before and after the reference time. There were no significant differences in the number of patients, patient recruitment periods, or patient characteristics between the two treatment groups. However, owing to the retrospective study design, we did not investigate the incidence of adverse events based on different RAI administration strategies. Another limitation is the difference of the follow-up periods according to RAI administration strategies. The follow-up period is significantly different because we applied different treatment strategies to patients on a specific time basis. Therefore, the follow-up period of patients treated with rhTSH plus 3.7 GBq is shorter than that of patients treated with THW plus 5.55 GBq (THW plus 150 mCi: 57.3 ± 20.1 months; rhTSH plus 100 mCi: 34.4 ± 9.6 months; *P* < 0.0001). However, participants in this study were patients with DTC patients with relatively poor prognosis (T4 or N1b disease), and recurrence was observed in about 8.7% of patients during follow-up. Nonetheless, long-term follow-up studies to evaluate prognosis in patients treated with these two approaches to adjuvant RAI therapy are needed, and our group is also planning a long-term follow-up study. Another limitation is the degree of TSH suppression during follow-up. Previous study reported a lesser degree of TSH suppression is associated with an increased incidence of relapse^19^. All patients included in the study, regardless of the treatment group, underwent TSH suppression according to the 2009 ATA guideline^10^. Therefore, the effect of prognosis on degree of TSH suppression in this study is expected to be minimal. Finally, this study did not include patients with microscopic or gross residual tumor tissue after surgery. Additional studies would be needed to apply these results to patients with microscopic or even gross residual disease.

## Conclusions

There were no differences in TSH-stimulated thyroglobulin level 6–18 months after adjuvant RAI therapy, rate of incomplete response after initial treatment, or RFS with the use of rhTSH plus 3.7 GBq instead of THW plus 5.55 GBq in patients with T4 or N1b disease. However, remnant uptake on diagnostic WBS was more frequently observed in patients who received rhTSH plus 3.7 GBq than in those who received THW plus 5.55 GBq, which resulted in significant difference in the rates of strict ablation success and rates of excellent response between the two groups. Adjuvant RAI therapy with rhTSH plus 3.7 GBq may be used instead of THW plus 5.55 GBq in DTC patients with T4 or N1b disease, although thyroid bed uptake on follow-up diagnostic WBS was more frequently observed. Further studies are needed to examine the clinical significance of the remnant uptake on diagnostic WBS after initial treatment.

## Supplementary information

Supplemental Table 1
