# Supplementary material for: Clinical outcomes of patients with T4 or N1b well-differentiated thyroid cancer after different strategies of adjuvant radioiodine therapy
Source: Sci Rep. 2019 Apr 3;9:5570. doi: 10.1038/s41598-019-42083-3 (PMC6447529; doi:10.1038/s41598-019-42083-3)
Supplement: Supplementary file 1 — Supplemental Table 1 [file 41598_2019_42083_MOESM1_ESM.pdf]

Clinical outcomes of patients with T4 or N1b well-differentiated thyroid cancer after different strategies of adjuvant radioiodine therapy

Shin Young Jeong[1,4], *Sang-Woo Lee[1,4], Wan Wook Kim[2,5], Jin Hyang Jung[2,5], Won Kee Lee[3], Byeong-Cheol Ahn[1,6], and Jaetae Lee[1,6]

[1]Department of Nuclear Medicine, [2]Department of Breast and Thyroid Surgery, [3]Medical Research Collaboration Center in KNUH, School of Medicine, Kyungpook National University, Daegu, Republic of Korea.

[4]Department of Nuclear Medicine, [5]Department of Breast and Thyroid Surgery, Kyungpook National University Chilgok Hospital, Daegu, Republic of Korea.

[6]Department of Nuclear Medicine, Kyungpook National University Hospital, Daegu, Republic of Korea.

Correspondence

Sang-Woo Lee, M.D., PhD., Professor and Director

Department of Nuclear Medicine, Kyungpook National University Chilgok Hospital

807 Hoguk-ro, Buk-gu, Daegu 41404, Republic of Korea

Tel: 82-53-200-2851, Fax: 82-53-200-2029, E-mail: swleenm@knu.ac.kr

Supplemental Table 1. Ablation success, diagnostic RAI whole body scan and classification of response to therapy after 5.55 GBq by THW and 3.7 GBq by rhTSH radioactive iodine therapy in old patients (>45 year old). Ablation success was determined using TSH-stimulated thyroglobulin level, neck USG findings, and diagnostic RAI whole body scan results. TSH-stimulated thyroglobulin measurement, neck USG, and diagnostic RAI whole body scan were performed at 6 to 18 months after adjuvant RAI therapy under levothyroxine withdrawal.

	THW plus 5.55 GBq n (%)	rhTSH plus 3.7 GBq n (%)	*P*-value
**Ablation Success**<br>s-Tg <1 ng/mL, negative TgAb<br>no suspicious finding on USG	45/67 (67.2%)	47/63 (74.6%)	0.351
**Ablation Success**<br>s-Tg <1 ng/mL, negative TgAb<br>no suspicious finding on USG<br>no residual thyroid bed uptake on diagnostic RAI WBS	41/67 (61.2%)	35/63 (55.6%)	0.514
**Diagnostic RAI WBS**			0.004
no uptake	60 (89.5%)	45 (71.4%)	
bed uptake	5 (7.5%)	18 (28.6%)	
pathologic uptake	2 (3.0%)	0 (0%)	
**Classification of Response to Treatment**			0.022
Excellent	39 (58.2%)	33 (45.8%)	
Indeterminate	17 (25.4%)	24 (38.1%)	
Biochemical Incomplete	5 (7.5%)	0 (0%)	
Structural Incomplete	6 (9.0%)	6 (9.5%)	

sTg: TSH-stimulated thyroglobulin

THW: thyroid hormone withdrawal

rhTSH: recombinant human thyrotropin

USG: neck ultrasonography

Diagnostic RAI WBS: diagnostic radioactive iodine whole-body scan
